# Insights into VTE risk in trauma patients: an observational study in an Irish trauma patient population

**DOI:** 10.1007/s11845-024-03866-4

**Published:** 2025-01-17

**Authors:** Bibi Ayesha Bassa, Elizabeth Little, Francis O. Keefe, Fionnuala Ní Áinle, Tomás Breslin, Valeria Lima Passos

**Affiliations:** 1 School of Postgraduate Studies, Royal College of Surgeons in Ireland, Dublin 2, Ireland; 2 Department of Trauma and Emergency Medicine, Mater Misericordiae University Hospital, Eccles Street, Dublin 7, Ireland; 3 Department of Haematology, Mater Misericordiae University Hospital, Eccles Street, Dublin 7, Ireland; 4 School of Pharmacy and Biomolecular Sciences (PBS), Royal College of Surgeons in Ireland, Dublin 2, Ireland

**Keywords:** Deep vein thrombosis (DVT), Pulmonary embolus (PE), Trauma, Venous thromboembolism (VTE)

## Abstract

**Background:**

The rate of VTE in trauma patients varies significantly in the reported literature. We aimed to determine the incidence of VTE in trauma patients in a trauma-receiving hospital over a 7-year period. We sought to evaluate the timing and nature of VTE events and explore the patterns of co-occurrence between PE and DVT, while factoring in clinical care and death outcome.

**Methods:**

Retrospective review of consecutive trauma patients ≥ 18 years admitted between January 2014 and December 2020. Data were extracted from the TARN database, picture archiving and communication system, and hospital records. The primary outcome was VTE incidence. Latent class analysis was used to uncover cross combinations of clinical management and VTE outcomes, yielding subgroups of trauma patients. Subgroups were compared for demographic and clinical characteristics.

**Findings:**

Seventy-three VTE were observed—incidence of 0.0036 cases/people-year (95% CI 0.0 to 3.69). VTE ( +) group consisted mostly of males (75%), had an advanced age, had higher injury severity scores, and had increased length of stay. Most patients (64%) developed a PE only. Most DVT (64%) were proximal. Two subgroups had a high probability of PE/low probability of DVT and two a high probability of DVT/low-to-moderate probability of PE. Subgroup comparisons showed differences in the clinical characteristics which were statistically inconclusive.

**Conclusion:**

This is the largest study of VTE incidence in Irish trauma patients and the first to delineate VTE risk in a trauma population. These findings urge reconsideration of VTE risk in trauma patients and implementation of prevention strategies.

**Supplementary Information:**

The online version contains supplementary material available at 10.1007/s11845-024-03866-4.

## Introduction

Venous thromboembolism (VTE), comprising deep vein thrombosis and pulmonary embolism, is a significant contributor to morbidity and mortality and affects more than one million people in Europe each year [1]. VTE is a leading cause of disability-adjusted life years (DALYs) lost and places significant financial burden on healthcare services [2]. Two-thirds of all VTE are hospital-acquired, occurring during or in the 90 days following hospitalisation [1, 3]. In hospitalised patients, trauma patients are among those with the highest risk of VTE; however, the reported rates of both PE and DVT vary significantly in the reported literature [4, 5]. Trauma is the leading cause of death in adults under 46 years, and the care of trauma patients accounts for more hospital days per year than the care of patients with cardiovascular disease or cancer [4]. In trauma patients who survive beyond initial resuscitation, PE is one of the leading causes of death [6].

The increased propensity to develop VTE in trauma patients is in part due to trauma-induced coagulopathy (TIC) [7]. TIC is characterised by two phases: an initial hypo-coagulable state resulting in uncontrolled bleeding, and a later hypercoagulable state, characterised by excessive micro- and macro-thrombosis and associated with VTE [7]. This occurs due to the physiological consequences of traumatic injuries including tissue injury, shock, endothelial dysfunction, coagulation, and immune upregulation, which are further exacerbated by acidosis and hypothermia [7].

While VTE is potentially preventable, thromboprophylaxis in trauma patients remains complex for two reasons: (i) a transient high risk of bleeding and (ii) a paucity of risk assessment models (RAM) and thromboprophylaxis guidelines validated in the trauma population. This picture is further exacerbated by complex injury patterns, increased operative needs, and continual invasive procedures. Moreover, the established concept of DVT and PE as a coupled entity in trauma patients has been probed, with studies suggesting that PE may occur de novo within the pulmonary arteries [8–10]. The combination of trauma-induced autonomic dysfunction, sympathetic upregulation, and endothelial wall inflammation is thought to promote local thrombus formation [10, 11]. These factors challenge decision-making in PE prevention and lead to uncertainty regarding timing, dosing, and duration of thromboprophylaxis post trauma.

Despite this, multiple decision-support tools, such as the Western Trauma Association critical decisions algorithm and the EAST practice management guidelines, have been developed to assist physicians in weighing the risk of bleeding against that of VTE [12, 13]. The diversity of decision-aids has resulted in significant practice variation in VTE prevention across hospitals and countries and may account for the disparities in VTE rates in trauma patients worldwide. In the republic of Ireland, there exists no national VTE guideline to assist trauma providers in determining the safe timing to initiate prophylaxis. VTE prophylaxis is at the discretion of attending consultants and local hospital policy. Current data on the incidence of VTE among our trauma population and its clinical relevance are lacking.

We sought to determine the incidence of VTE in a large Irish trauma-receiving hospital over a 7-year period. Further, we aimed to evaluate the timing and nature of VTE events and explore the patterns of co-occurrence between PE and DVT, while factoring in clinical care and death outcome. The findings of this study will inform strategies to improve our standard of care in preventing VTE in our trauma population.

## Methods

This study was conducted in the Department of Emergency Medicine of the Mater Misericordiae University Hospital (MMUH). MMUH is a level 4 teaching institution, a trauma-receiving hospital for the central trauma network and the National Spinal Unit in Ireland. We conducted a retrospective review of consecutive major trauma patients ≥ 18 years admitted to MMUH between the years of January 2014 and December 2020.

### Study population

All adult major trauma patients presenting to MMUH during the study period and eligible for inclusion in the TARN registry were included. TARN is a national clinical audit body for traumatic injury that captures trauma data of affiliated members to a central trauma registry [14]. A total of 353,859 patients presented to the MMUH emergency department between the years of January 2014 and December 2020, of which 2920 (0.83%) were major trauma patients.

### Inclusion criteria

Trauma patients ≥ 18 years that met criteria for entry into the TARN database during the study period were included [14]. This included the following:

- Length of stay > 3 days
- Required admission to critical care medicine
- Deaths of trauma patient occurring in the hospital
- Transfer of patient into and out of MMUH for specialist care

### Exclusion criteria

All trauma patients < 18 years and patients transferred outside of Ireland were excluded from this study. Patients with incomplete or missing data TARN data were also excluded.

### Data sources

Data were collected from the TARN database, the MMUH digital radiology Picture Archiving and Communication System (PACS), and inpatient chart reviews.

Using the TARN database, data were collected retrospectively for the period 01/01/2014–31/12/2020 by resident TARN coordinator. Patient submission identifiers were cross-matched with medical record numbers to extract data from the National Integrated Medical Imaging System (NIMIS) to review imaging investigating VTE during and up to 90 days post admission. Patient chart reviews were conducted for patients with radiologically confirmed VTE.

Data collected included age, biological sex, mechanism of injury (MOI), length of hospital stay (LOS), injury severity score (ISS), and VTE event (PE and/or DVT) during and up to 90 days post admission. Diagnoses of VTE were in line with the International Society on Thrombosis and Haemostasis (ISTH) recommendations [15]. DVT was defined as thrombus identified on either lower limb duplex Doppler ultrasound or computed venography. PE was defined as a filling defect within the pulmonary arterial system identified on any form of contrast-enhanced thoracic computed imaging.

VTE were categorised as immediate (present on admission), early (< 48 h of admission), or late (48 h to 90 days post admission) [16]. We further analysed variables of patients with confirmed VTE including primary injury, anatomical location of DVT and/or PE, evidence of completed VTE risk assessment tools, prophylaxis (mechanical and/or chemical), and mortality.

### Statistical analysis

#### The general trauma patient sample

Descriptive statistics were used to summarise clinical and demographic characteristics. Categorical variables were presented as percentages and absolute counts. Continuous variables were shown as means and standard deviations or medians and IQR, where appropriate. For comparisons between patients with VTE versus non-VTE patients, Student’s *t*-test and/or Mann–Whitney test was used for continuous variables, and chi-square test was used for categorical variables. Statistical analyses were performed using 9.4 (SAS Institute, Cary, NC, USA).

#### Patients with radiologically confirmed VTE (“VTE ( +)”)

##### Latent class analysis

Latent class analysis is a person-centred, model-based clustering technique used to unveil different (not directly observable) subgroups within a population that share certain characteristics, allowing more detailed investigation of underlying heterogeneity in clinical patterns [17]. In this study, latent class analysis (LCA) was used to extract data-driven subgroups of trauma patients who shared similar inpatient clinical management and VTE outcomes.

##### LCA indicators

Based on internal VTE audits and clinical insight of resident VTE working group, characteristics of trauma patients repeatedly developing VTE were identified as LCA indicators. These included surgery within 48 h of admission (yes/no), intubation on admission (yes/no), admission to intensive care unit (ICU) (yes/no), need for an IVCF filter (yes/no), and death outcome [18–23]. VTE outcome variables included DVT or PE (yes/no).

##### LCA model selection

Determining the final number of latent classes was assisted by model fit criteria (Akaike information criterion (AIC), consistent Akaike information criterion (CAIC), adjusted Bayesian information criterion (BIC)), while weighing interpretability and visual inspection of the classes’ distinctiveness. Entropy was used as an index for classification accuracy.

##### Latent class profiling

Following a review of the current literature assessing risk factors for VTE in trauma, risk factors found to be independently associated with VTE in trauma were considered for comparisons among extracted latent classes. Demographics included age and biological sex [18, 19]. Trauma registry variables included ISS, LOS, and MOI [20]. Injury variables included primary injury [20, 21]. VTE variables included VTE type (immediate, early, or late), evidence of completed risk assessment, and time to prophylaxis. Comparisons were conducted with the either ANOVA or its non-parametric alternative for continuous variables, and Fisher’s exact test for categorical variables.

This study was approved by the Mater Misericordia University Hospital Institutional Review Board (IRB Ref: 1/378/2333).

## Results

### General trauma population

A total of 2920 trauma patients presented to MMUH for the period 01/01/2014 to 31/12/2020 and were included in the TARN database. Of these patients, 21 were transferred out of Ireland and 20 had incomplete data captured on the TARN database. Therefore, 2879 patients were included in the study.

During January 2014–December 2020, 73 VTE events were recorded. These data suggest a VTE incidence of 0.0036 cases/people-year (95% CI 0.0–3.69), or 3.6 cases/1000 people-year. Male patients accounted for roughly two-thirds of the total trauma population but constituted almost three quarters of the VTE ( +) group (*p*-value = 0.0572) (risk ratio (RR) 1.66; 95% CI 0.98–2.82). There were a substantially larger proportion of deaths in the VTE ( +) compared to the non-VTE group (16.4% vs. 9.51%) correlating to a RR of 1.63 (95% CI 0.95–2.78). The patient characteristics of the trauma population are shown in Table 1.

**Table 1:** Patient characteristics of trauma population

	Total trauma population (*n* = 2879)	Non-VTE group (*n* = 2806)	VTE ( +) group (*n* = 73)	*P*-value
Age (mean, SD)	60 (22)	61 (22)	63 (18)	0.1386
Injury Severity Score (ISS) (median, IQR)	10 (10)	10 (10)	13.5 (16)	0.0177
Length of stay (LOS) (median, IQR)	8 (13)	9 (15)	16.5 (42)	< 0.0001
Biological sex (*N*) (%)				0.0572
Male	1856 (64)	1801 (64)	55 (75)	
Female	1023 (35)	1005 (36)	18 (25)	
Mechanism of injury (*N*) (%)				0.6105
Blows without weapons	242 (8.40)	240 (8.5)	2 (3)	
Road traffic accident (RTA)	14 (0.5	2 (0.10)	12 (16)	
Vehicle collision	542 (19)	540 (19)	2 (3)	
Fall < 2 m	1444 (50)	1400 (50)	44 (60)	
Crush injury	14 (0.50)	12 (0.40)	2 (3)	
Fall > 2 m	464 (16)	454 (16)	10 (14)	
Shooting	15 (0.50)	15 (0.50)	-	
Stabbing	62 (2)	62 (2)	-	
Blast	4 (0.10)	4 (0.10)	-	
Burn	10 (0.30)	10 (0.30)	-	
Assault	69 (2)	69 (2.)	-	
Other	2 (0.10)	2 (0.10)	-	
VTE events (*N*) (%)	73 (2.55)	0	73	
DVT only	21 (0.7)	-	21 (29)	
PE only	47 (2)	-	47 (64)	
DVT + PE	5 (0.2)	-	5 (7)	
Mortality (deaths) (%)	279	267 (9.5)	12 (16.4)	0.0549

Most patients (64%) developed a PE only. Fewer than 10% developed both DVT and PE. An overwhelming majority of DVT events (> 85%) occurred later. Nearly two-thirds of all PE events (62%) occurred late (see Tables 2 and 3). The majority of DVT were proximal (64%). Upper limb DVT accounted for just over 15% of DVT events. Segmental PE accounted for roughly half of all PE diagnoses. A significant proportion of PE (30%) involved the subsegmental branches of the pulmonary vasculature. Four patients demonstrated evidence of right ventricular strain on CT (see Tables 4 and 5).

**Table 2:** Summary of VTE events

VTE events (*n* = 73)	
DVT only (*N*) (%)	21 (29)
PE only (*N*) (%)	47 (64)
DVT + PE (*N*) (%)	5 (7)
Timing of VTE events	
Immediate (on admission) (*N*) (%)	10 (13.50)
Early (< 48 h) (*N*) (%)	14 (19)
Late (> 48 h) (*N*) (%)	49 (67)

**Table 3:** Breakdown of PE and DVT events by timing

	Immediate	Early	Late
DVT (*N*) (% of total DVT events)	1 (3.85)	2 (7.70)	23 (88.5)
PE (*N*) (% of total PE events)	9 (17)	11 (21)	32 (62)

**Table 4:** Anatomical location of pulmonary emboli (PE)

Pulmonary embolism		
	*N*	%
Main arteries	7	13
Lobar	10	18.50
Segmental	27	50
Subsegmental	16	30
Evidence of right ventricular (RV) strain	4	7.40

**Table 5:** Anatomical location of deep vein thromboses (DVT)

Deep vein thrombosis		
	N	%
Upper limb	4	16.70
Inferior vena cava (IVC) extending distally	2	7.70
Internal and external Iliac veins	4	16.70
Common femoral vein	4	16.70
Femoropopliteal vein	6	23
Below knee	1	3.85

### Latent class analysis *—* ‘VTE ( +)*’ *patients only

**Fig. 1:**
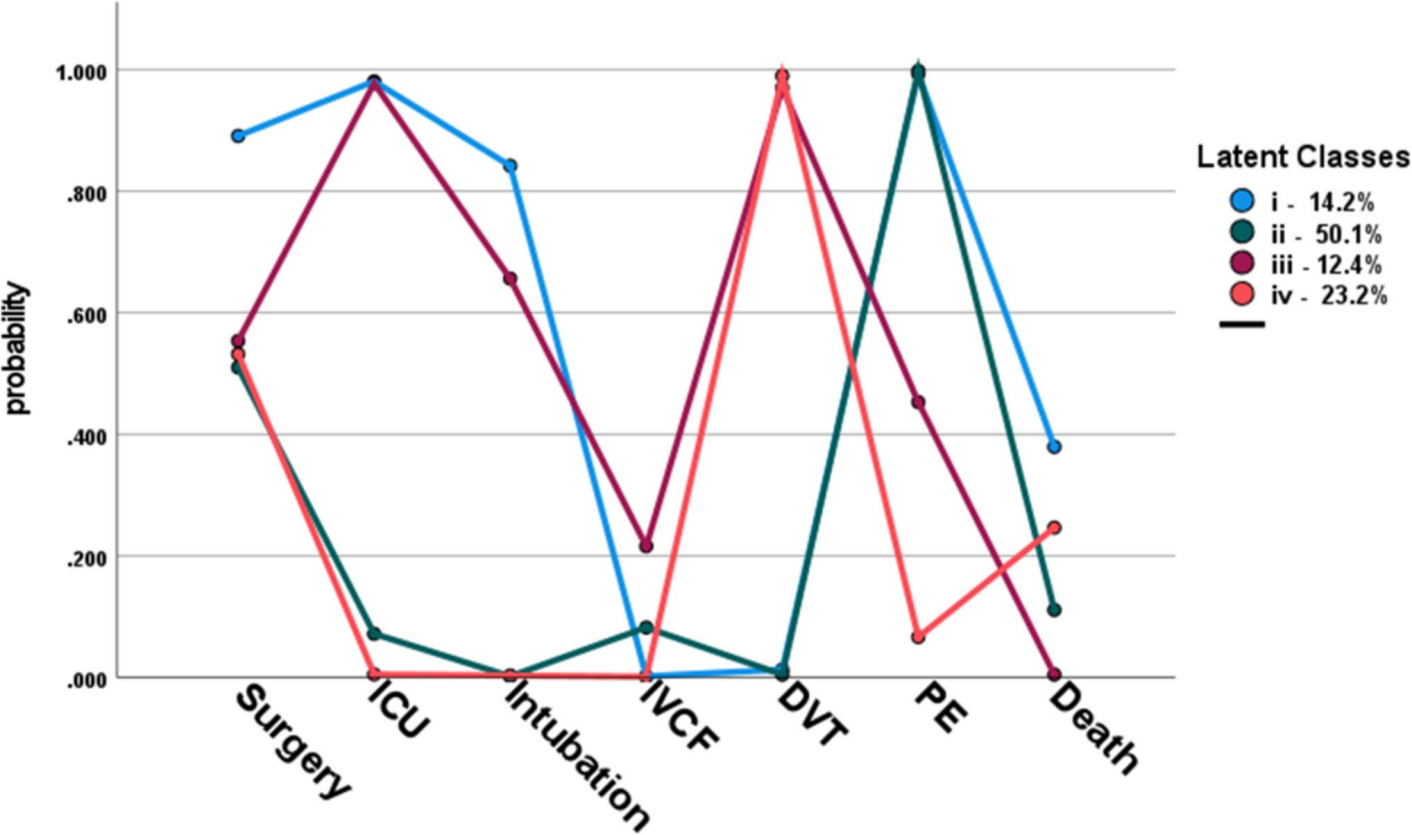
Item probability plot showing the four subgroups (with estimated sizes) extracted by LCA. These groups are characterised by distinct cross-combinations among the clinical care course variables and VTE outcomes. The *y*-axis captures the probability of patients to have experienced the selected indicators (*x*-axis) within each of the four subgroups. Intensive care unit (ICU), inferior vena cava filter (IVCF), deep vein thrombosis (DVT), pulmonary embolism (PE)

We fitted LCA models with two up to six latent classes. The associated fit criteria, displayed in Table 2 of the supplemental material, supported models with three or four classes. We settled for the four-class solution as it yielded well-discriminated and clinically meaningful profiles (see Fig. 1 of supplemental material). The main features of the extracted four classes, capturing the heterogeneity in cross-combinations of the selected indicators, are displayed in the item probability plot (Fig. 1). This plot shows the probability of patients (*y*-axis) to have experienced the selected outcomes (*x*-axis) within each of the four subgroups. Accordingly, these were named after their distinguishing clinical course: (i) moderate to high-risk trauma patients requiring critical care, (ii) low-risk uncomplicated trauma patients, (iii) nil risk trauma patients requiring critical care, and (iv) moderate risk uncomplicated trauma patient. Risk refers to the class mortality. Sociodemographic and additional clinical descriptives across the latent classes and results of their comparisons are shown in Table 6.

**Table 6:** Description of sociodemographic and additional clinical characteristics across the latent classes and the results of class comparisons

	**i** Mod–high-risk trauma requiring critical care	**ii** Low-risk uncomplicated trauma	**iii** Nil risk trauma requiring critical care	**iv** Mod risk uncomplicated trauma	*P*-value
*N* (%)	9 (12)	38 (52)	9 (12)	17 (23)	
Age (Mean, SD)	58 (16)	67 (15)	52 (19)	61 (22)	0.1100
Biological sex (*N*) (%)					0.1366
Male	8 (89)	25 (65)	9 (100)	13 (76)	
Female	1 (11)	13 (35)	0	4 (24)	
Injury Severity Score (ISS) (median, IQR)	17 (20)	13 (16)	25 (22)	11.5 (14.5)	0.1325
Length of stay (LOS) (median, IQR)	33 (65)	14 (23)	32 (71)	17 (42.5)	0.1908
Mechanism of injury (*N*) (%)					0.3001
Blows without weapons	-	2 (5)	1 (11)	-	
Road traffic accident (RTA)	2 (22)	6 (16)	3 (33)	1 (6)	
Vehicle collision	-	-	4 (45)	2 (12)	
Fall < 2 m	3 (33)	25 (66)	-	11 (65)	
Crush injury	1 (11)	-	-	1 (6)	
Fall > 2 m	3 (33)	4 (11)	1 (11)	2 (12)	
Other	-	1 (3)	-	-	
Primary injury (*N*) (%)					0.5936
Blunt abdominal injury	-	2 (5)	-		
Chest wall injury	-	2 (5)	2 (22)	2 (12)	
Isolated femur fracture	1 (11)	7 (18)	1 (11)	1 (6)	
Isolated neck of femur (NOF) fracture	1 (11)	2 (5)	-	-	
Isolated tibia-fibula fracture	-	1 (3)	-	1 (6)	
Pelvic fracture	1 (11)	1 (3)	-	-	
Traumatic urethral Injury		-	-	-	
Polytrauma	1 (11)	4 (11)	1 (11)	2 (12)	
Subdural haemorrhage (SDH)/subarachnoid haemorrhage (SAH)	-	6 (16)	1 (11)	3 (18)	
Spinal fracture	5 (56)	13 (34)	2 (22)	5 (29)	
VTE type (*N*) (%)					0.0580
Immediate VTE	2 (22)	7 (18)	0	1 (6)	
Early VTE	4 (45)	8 (21)	0	2 (12)	
Late VTE	3 (33)	23 (60)	9 (100)	14 (82)	
VTE risk assessment (RA)					
VTE RA completed (*N*) (%)	6 (67)	20 (52)	5 (55)	10 (59)	0.7661
Received VTE prophylaxis (*N*) (%)	4 (44)	20 (52)	5 (55)	10 (59)	0.2412
Hours to start VTE prophylaxis (median; IQR)	72 (96)	60 (144)	24 (120)	24 (144)	0.2177

Two groups, *i* moderate to high-risk trauma requiring critical care and *ii* low-risk uncomplicated trauma, had a high probability of developing a PE without having an initial DVT. Group *i* consisted of complex trauma patients who underwent surgical intervention and ICU admission—thus intensive care management—and, though moderate, had the highest probability of death. This group represents the most critical trauma patients, who had the longest LOS and developed PE only. Traumatic spinal injury was particularly prevalent in this group. This group had the highest proportion of completed risk assessments but the longest median time (longer than 72 h) to start mechanical or chemical thromboprophylaxis.

Circa half of the patients in group *ii* (the largest group, constituting half of all the VTE + cases) required surgery, but were less likely to require ICU admission. Despite their uncomplicated course, this group developed PE, but, in comparison to group *i*, had a lower death risk. Nearly two-thirds of PE occurred in this group were late. The majority of femur fractures and traumatic brain injury (TBI) occurred in this group.

Patients in groups *iii*, nil risk trauma requiring critical care, and *iv*, moderate risk uncomplicated trauma, had DVT, but only the former had a moderate PE risk (ca. ~ 40% of the patients). In both groups, more than half of them underwent surgery and had the shortest median time to start thromboprophylaxis (24 h). A key distinction between *iii* and *iv* was care management, with patients in group *iii* requiring critical care admission, intubation, and a small proportion IVCF insertion, but no deaths were reported. By contrast, group *iv* had a higher proportion of deaths, though still low. This group had the highest likelihood of developing both a DVT and PE, of which all were late events, and was made up exclusively by men, who had the youngest median age.

Of note, all comparisons among the latent classes were statistically inconclusive (at the 5% significant level), most likely due to the small sample sizes.

## Discussion

This study is the first to evaluate and explore the co-occurrence of VTE outcomes and trauma care characteristics in an Irish trauma population. Majority of studies investigating VTE in trauma patients originate outside of Europe with few reporting VTE incidence in the European trauma population. During the 7-year period, VTE incidence in our trauma population was 0.0036 cases/people-year (95% CI 0.0 to 3.69). Globally, the estimated rates of VTE in trauma patients range from 0.39 to 11.20% [22, 24]. This variation is mirrored in both DVT rates (0.59 to 31.90%) and PE rates (0.32 to 6.8%) [25, 26]. These differences are largely due to patient demographics, VTE surveillance strategies, reporting of VTE outcomes, and institutional VTE thromboprophylaxis protocols [27].

Despite our low VTE incidence, we were able to parse the heterogeneity of DVT and PE outcomes and associated clinical management, and explore possible links between identified sub-groups, VTE timing, and individual clinical characteristics. Two groups had a *high probability of PE and low probability of DVT* and two had a *high probability of DVT but a low to moderate probability of PE*. Additionally, we have found that most PE events occurred independently of DVT with less than 10% of patients developing both concurrent DVT and PE.

### Trauma patients with high probability to develop DVT

In our trauma population, we had a DVT rate of 0.001% per annum with roughly 65% occurring proximally. These DVT were diagnosed in patients with clinical signs suggestive of DVT. None was detected as part of routine surveillance. Evidence has shown that surveillance bias tends to identify more distal DVT, does not reduce the risk of PE or fatal PE, and leads to unnecessary anticoagulation due to false positive finding [12, 28]. Distal DVT do have the same prognostic significance as proximal DVT due to lower risk of embolisation, with studies suggesting that 80% resolve spontaneously [29].

In our study, groups *iii*, *nil risk trauma patients requiring critical care*, and *iv*, *moderate risk uncomplicated trauma patients*, had a high likelihood of developing DVT only. Both groups underwent surgery within 48 h of admission (more than the half of all patients). This finding is partly consistent with data suggesting a significant association between DVT and surgery in trauma patients, with the risk of thrombosis starting perioperatively [19, 30, 31]. Across groups iii and iv, spinal injury and intracranial haemorrhage occurred in over 50% of patients. Neurosurgery and spinal surgery remain absolute indications for withholding thromboprophylaxis, with the optimal timing to restart postoperatively ranging between 24 and 72 h [32]. Delayed initiation of prophylactic LMWH 12 to 24 h postoperatively has been shown to result in suboptimal antithrombotic effectiveness [33]. Reiff and colleagues [34] demonstrated a three- to fourfold increase in DVT risk in patients with TBI. DVT has been found to occur in one-third of moderate and severe patients with isolated head injuries [35]. Knudson et al. [30] reported patients with major head injury to be more at risk for DVT (OR, 1.34) than for PE (OR, 0.87). Patients with traumatic spinal injuries have an increase VTE risk which is often outweighed by the risk of epidural haematoma expansion [12, 13]. Groups *iii* and *iv* reflect a potential description of trauma patients with *high probability to develop DVT* who may benefit from targeted DVT surveillance and prevention.

### Trauma patients with high probability to develop PE

In our setting, we reported a PE incidence of 0.002% per annum. In their review, Shuster et al. [8] determined the rates of post trauma PE to be variable and dependent on study design, inclusion, and diagnostic criteria. Differences in PE rates in trauma patients have been attributed to advances in CT technology, 24-h availability of CT scanners, and liberal use of immediate imaging by trauma centres [30, 36]. In a UK major trauma centre study, Glover et al. [37] found a PE rate of 4.6% in their trauma population, with most PEs occurring after 72 h of admission. This is similar to our finding of late PE accounting for 62% of all PE events.

In our most critically unwell trauma patients (group *i — Moderate-high risk trauma requiring critical care*), nearly two-thirds of all PE events occurred within 48 h of admission and in the absence of DVT. Benns et al. found a significant number of PEs occurring early in the hospital course, with no prior DVT, and concluded that PE occurring early is likely secondary to biochemical mechanisms outside the conventional explanation of distal clot embolisation [11]. In investigating predictors of early versus late PE in trauma patients, Velmahos et al. [10] found that most PE patients did not have evidence of prior DVT and suggested that certain PE may occur de novo within the lungs. Early PE may potentially arise due to local injury and TIC, with later PE a result of embolisation of distal thrombus caused by prolonged immobility and inadequate anticoagulation [8]. Brakenridge et al. [16] found that half of all PEs in their trauma population occurred within the first 4 days after injury, and that patients with severe head injuries were more likely to experience late PE. Gambhir et al. [38] found proximal DVT as the largest risk factors for late PE. This is a crucial finding to this study that in our population, the most critically unwell trauma patients are more likely to develop early PE without any preceding indication of a DVT. In an already vulnerable group, this can significantly compound recovery.

### Interpretation

What this study has enabled us to observe, is that, in trauma patients, DVT and PE can occur both concurrently and independently of each other. As opposed to evaluating the contribution of each risk factor individually, we have considered VTE risk as multidimensionally determined, identifying their patterns of co-occurrence with trauma management variables, and exploring their links to types of injury and patients’ characteristic. This is important, as VTE risk in trauma patients has been associated with both individual patient characteristics (age, biological sex, history of VTE, etc.) and interventions carried out as part of their acute trauma resuscitation (femoral venous line insertions, mechanical ventilation, major operative repair, etc.). Therefore, the authors acknowledge that the cause of VTE in trauma patients is multifactorial.

Identifying trauma patients with higher DVT and PE risk can facilitate earlier discussions on thromboprophylaxis strategies, potential surveillance screening, and the use of inferior vena cava filters. This approach to risk, as opposed to a generalisable standard, can enable clinicians to understand the risk hierarchy of PE and DVT and focus DVT and PE prevention practice in accordance with injury and management characteristics.

### Limitations

This study is not without limitations. Due to the retrospective nature, we were limited to a small sample which affected the statistical power of the conducted comparisons and the generalisability of our findings. As part of our methodology, this study utilised the TARN database and inpatient chart records for data collection. Missing data (due to incomplete patient capturing) in the TARN database limited our sample. Due to the lack of a national electronic health record system in Ireland, it is possible that patients may have represented post discharge to hospitals outside of the NIMIS PACS network, resulting in their omission from the VTE ( +) group. In addition, the low rates of routine post-mortem examination may underestimate the incidence of VTE. Lastly, although our hospital has a local VTE guideline, decisions on VTE prophylaxis in trauma patients are at the admitting physician’s discretion. This may have affected the VTE outcomes in our population.

### Implications

Across major trauma centres, including the UK, decision-making with respect to the timing of VTE prophylaxis in trauma patients is often made by multidisciplinary teams on a case-by-case basis [39]. Individual patient characteristics continue to guide the safe selection and timing of prophylaxis, with daily VTE and bleeding risk assessment recommended as the standard of care [39, 40]. What this study highlights is the need for trauma-specific, VTE prevention guidelines. Understanding that trauma patients are often complex, a support tool can help clinicians set early and safe target times to prophylaxis. The centralised availability of information addressing various injury patterns can assist with the standardisation of VTE prevention practice in Ireland. The national VTE program (NTVEP) is best positioned to provide the clinical and administrative support to supervise the development of such a guideline.

## Conclusion

We have a VTE incidence of 0.0036 cases/people-year (95% CI 0.0 to 3.69) in our trauma population, with PE occurring with and without DVT in trauma patients. Heterogeneity exists in the nature and timing of VTE events in trauma patients and can potentially underlie unveiled patterns of co-occurrence between clinical care and death outcome. Overall, these findings provide a compelling argument for the implementation of VTE strategies specific to trauma populations as well as for the development of an objective risk assessment model for VTE post trauma. A greater understanding of the exact mechanism of PE formation post trauma requires further investigation to better inform PE prevention.

## Supplementary Information

Below is the link to the electronic supplementary material.

Supplementary file1 (DOCX 123 KB)

Supplementary file2 (DOCX 97 KB)

Supplementary file3 (DOCX 152 KB)

## Data Availability

All data produced or analysed during this study are included in this published article.
