# Supplementary material for: Insights into VTE risk in trauma patients: an observational study in an Irish trauma patient population
Source: Ir J Med Sci. 2025 Jan 17;194(1):195–204. doi: 10.1007/s11845-024-03866-4 (PMC11861230; doi:10.1007/s11845-024-03866-4)
Supplement: Supplementary file 1 — Supplementary file1 (DOCX 123 KB) [file 11845_2024_3866_MOESM1_ESM.docx]

**Supplementary Material**

Table 1: VTE risk assessment and prophylaxis strategies used in VTE (+) group

| VTE (+) group (n = 73) | | |
| --- | --- | --- |
|  | N | % |
| Evidence of completed risk assessment on admission | 41 | 55 |
| Mechanical prophylaxis used |  |  |
| - Thromboembolic deterrent stockings (TEDS) | 20 | 27 |
| - Intermittent pneumatic compression (IPC) | 2 | 3 |
| Chemical prophylaxis used |  |  |
| - Enoxaparin | 15 | 20 |
| - Clexane | 6 | 8 |
| - Direct oral anticoagulant (DOAC) | 5 | 7 |
| Inferior vena cava filter (IVCF) inserted | 5 | 8 |
| No prophylaxis used | 3 | 4 |
| Documented contraindication for cases with no chemical prophylaxis: | | |
| Active bleeding | 2 | 3 |
| High risk intracranial haemorrhage | 2 | 3 |
| Dropping haemoglobin (Hb) levels | 2 | 3 |
| Blood transfusion requirements | 1 | 1.5 |
| Intra-operative cerebrovascular accident (CVA) | 1 | 1.5 |
| Spontaneous thrombocytopenia | 1 | 1.5 |
| Active cancer for palliation | 1 | 1.5 |

Table 2: Associated Fit Criteria for 3 and 4 class solution

| 2 | 3 | 4 | 5 | 6 |
| --- | --- | --- | --- | --- |
| Log-likelihood: 251.45  AIC: 140.53  BIC: 175.09  CAIC: 190.09  Adjusted BIC: 127.82  Entropy: 0.92 | Log-likelihood: 227.21  AIC: 108.05  BIC: **161.04**  CAIC: **184.04**  Adjusted BIC: 88.56  Entropy: 0.95 | Log-likelihood: -216.21  AIC: **102.04**  BIC: 173.46  CAIC: 204.46  Adjusted BIC: **75.77**  Entropy: 0.96 | Log-likelihood: -212.29  AIC: 110.21  BIC: 200.07  CAIC: 239.07  Adjusted BIC: 77.16  Entropy: 0.96 | Log-likelihood: -208.80  AIC: 119.22  BIC: 227.51  CAIC: 274.51  Adjusted BIC:79.40  Entropy: 0.96 |

Class: **1** **2** **3** **4**

**0.1442 0.5043** **0.1226** **0.2289**

(0.0575) (0.0700) (0.0391) (0.0491)


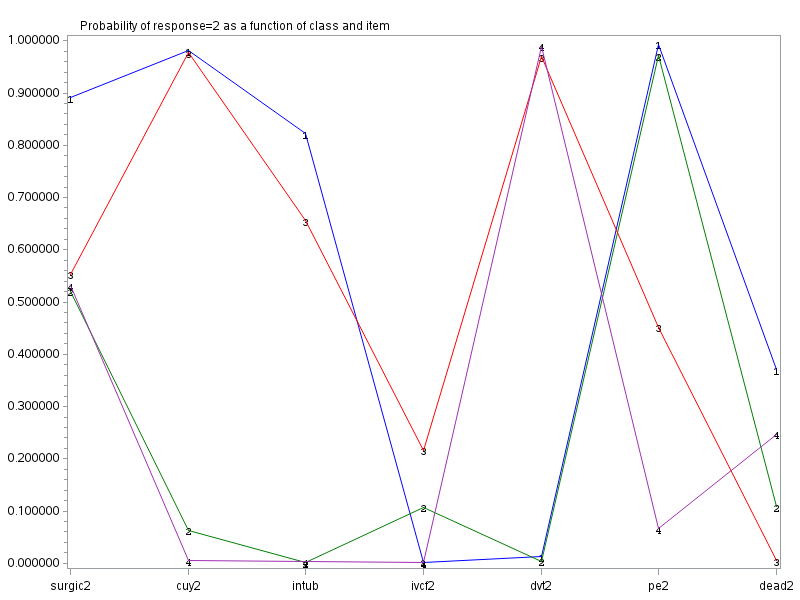


Figure 1: Item probability plot - K= 4 solution

In the interest of transparency, the k=3 solution, which was also a good fit to the data (CAIC and BIC), is presented below. Notice that in the transition from 3 to 4 solutions, class 2 (k=3 solution) was separated into two classes (1 & 3 in the k=4 solution), both yielding clinically relevant and well-discriminated sub-groups. This change had the potential to enhance interpretability was further supported by the fit criteria (AIC and Adjusted BIC).

Class: 1 2 3

0.4972 0.2739 0.2289

(0.0649) (0.0591) (0.0492)


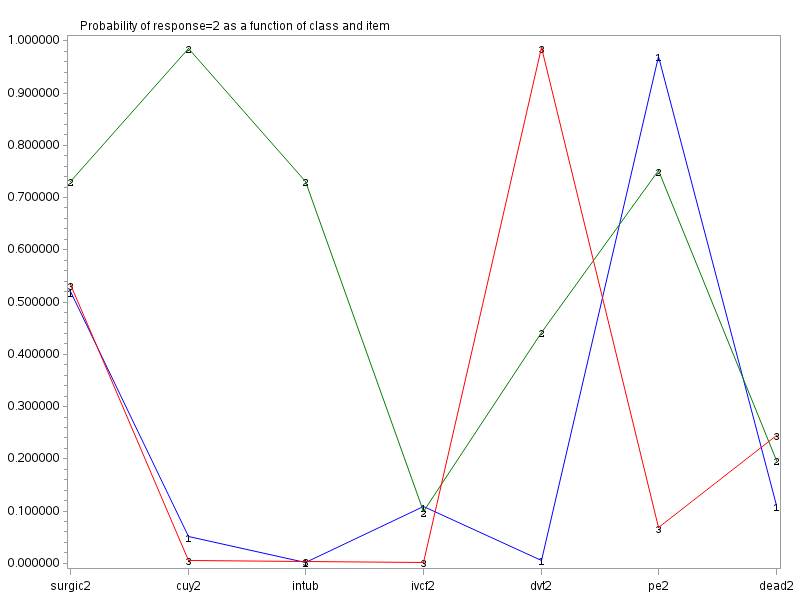


Figure 2: Item probability plot - K= 3 solution
