# Supplementary material for: Insights into VTE risk in trauma patients: an observational study in an Irish trauma patient population
Source: Ir J Med Sci. 2025 Jan 17;194(1):195–204. doi: 10.1007/s11845-024-03866-4 (PMC11861230; doi:10.1007/s11845-024-03866-4)

**Supplementary Material**

| The SAS System |
| --- |

The FREQ Procedure

| \| \| **Frequency** \| \| --- \| \| **Percent** \| \| **Row Pct** \| \| **Col Pct** \| \| \| --- \| --- \| --- \| --- \| --- \| | \| **Table of gender by Final_group** \| \| \| \| \| \| \| --- \| --- \| --- \| --- \| --- \| --- \| \| **gender(Gender)** \| **Final_group(Final_group)** \| \| \| \| \| \| **1** \| **2** \| **3** \| **4** \| **Total** \| \| **Male** \| \| 8 \| \| --- \| \| 10.96 \| \| 14.55 \| \| 88.89 \| \| \| 25 \| \| --- \| \| 34.25 \| \| 45.45 \| \| 65.79 \| \| \| 9 \| \| --- \| \| 12.33 \| \| 16.36 \| \| 100.00 \| \| \| 13 \| \| --- \| \| 17.81 \| \| 23.64 \| \| 76.47 \| \| \| 55 \| \| --- \| \| 75.34 \| \|  \| \|  \| \| \| **Female** \| \| 1 \| \| --- \| \| 1.37 \| \| 5.56 \| \| 11.11 \| \| \| 13 \| \| --- \| \| 17.81 \| \| 72.22 \| \| 34.21 \| \| \| 0 \| \| --- \| \| 0.00 \| \| 0.00 \| \| 0.00 \| \| \| 4 \| \| --- \| \| 5.48 \| \| 22.22 \| \| 23.53 \| \| \| 18 \| \| --- \| \| 24.66 \| \|  \| \|  \| \| \| **Total** \| \| 9 \| \| --- \| \| 12.33 \| \| \| 38 \| \| --- \| \| 52.05 \| \| \| 9 \| \| --- \| \| 12.33 \| \| \| 17 \| \| --- \| \| 23.29 \| \| \| 73 \| \| --- \| \| 100.00 \| \| |
| --- | --- | --- | --- | --- | --- | --- | --- | --- | --- | --- | --- | --- | --- | --- | --- | --- | --- | --- | --- | --- | --- | --- | --- | --- | --- | --- | --- | --- | --- | --- | --- | --- | --- | --- | --- | --- | --- | --- | --- | --- | --- | --- | --- | --- | --- | --- | --- | --- | --- | --- | --- | --- | --- | --- | --- | --- | --- | --- | --- | --- | --- | --- | --- | --- | --- | --- | --- | --- | --- | --- | --- | --- | --- | --- | --- | --- | --- | --- | --- | --- | --- | --- | --- | --- | --- | --- | --- | --- | --- | --- | --- |

| **Fisher's Exact Test** | |
| --- | --- |
| **Table Probability (P)** | 0.0021 |
| **Pr <= P** | 0.1366 |
|  |  |

**Sample Size = 73**

| \| \| **Frequency** \| \| --- \| \| **Percent** \| \| **Row Pct** \| \| **Col Pct** \| \| \| --- \| --- \| --- \| --- \| --- \| | \| **Table of prim_inj by Final_group** \| \| \| \| \| \| \| --- \| --- \| --- \| --- \| --- \| --- \| \| **prim_inj(prim_inj)** \| **Final_group(Final_group)** \| \| \| \| \| \| **1** \| **2** \| **3** \| **4** \| **Total** \| \| **Other** \| \| 2 \| \| --- \| \| 2.74 \| \| 10.53 \| \| 22.22 \| \| \| 11 \| \| --- \| \| 15.07 \| \| 57.89 \| \| 28.95 \| \| \| 2 \| \| --- \| \| 2.74 \| \| 10.53 \| \| 22.22 \| \| \| 4 \| \| --- \| \| 5.48 \| \| 21.05 \| \| 23.53 \| \| \| 19 \| \| --- \| \| 26.03 \| \|  \| \|  \| \| \| **Spinal Injury** \| \| 5 \| \| --- \| \| 6.85 \| \| 20.00 \| \| 55.56 \| \| \| 13 \| \| --- \| \| 17.81 \| \| 52.00 \| \| 34.21 \| \| \| 2 \| \| --- \| \| 2.74 \| \| 8.00 \| \| 22.22 \| \| \| 5 \| \| --- \| \| 6.85 \| \| 20.00 \| \| 29.41 \| \| \| 25 \| \| --- \| \| 34.25 \| \|  \| \|  \| \| \| **Femur fractures** \| \| 1 \| \| --- \| \| 1.37 \| \| 10.00 \| \| 11.11 \| \| \| 7 \| \| --- \| \| 9.59 \| \| 70.00 \| \| 18.42 \| \| \| 1 \| \| --- \| \| 1.37 \| \| 10.00 \| \| 11.11 \| \| \| 1 \| \| --- \| \| 1.37 \| \| 10.00 \| \| 5.88 \| \| \| 10 \| \| --- \| \| 13.70 \| \|  \| \|  \| \| \| **TBI** \| \| 0 \| \| --- \| \| 0.00 \| \| 0.00 \| \| 0.00 \| \| \| 6 \| \| --- \| \| 8.22 \| \| 42.86 \| \| 15.79 \| \| \| 3 \| \| --- \| \| 4.11 \| \| 21.43 \| \| 33.33 \| \| \| 5 \| \| --- \| \| 6.85 \| \| 35.71 \| \| 29.41 \| \| \| 14 \| \| --- \| \| 19.18 \| \|  \| \|  \| \| \| **Polytrauma** \| \| 1 \| \| --- \| \| 1.37 \| \| 20.00 \| \| 11.11 \| \| \| 1 \| \| --- \| \| 1.37 \| \| 20.00 \| \| 2.63 \| \| \| 1 \| \| --- \| \| 1.37 \| \| 20.00 \| \| 11.11 \| \| \| 2 \| \| --- \| \| 2.74 \| \| 40.00 \| \| 11.76 \| \| \| 5 \| \| --- \| \| 6.85 \| \|  \| \|  \| \| \| **Total** \| \| 9 \| \| --- \| \| 12.33 \| \| \| 38 \| \| --- \| \| 52.05 \| \| \| 9 \| \| --- \| \| 12.33 \| \| \| 17 \| \| --- \| \| 23.29 \| \| \| 73 \| \| --- \| \| 100.00 \| \| |
| --- | --- | --- | --- | --- | --- | --- | --- | --- | --- | --- | --- | --- | --- | --- | --- | --- | --- | --- | --- | --- | --- | --- | --- | --- | --- | --- | --- | --- | --- | --- | --- | --- | --- | --- | --- | --- | --- | --- | --- | --- | --- | --- | --- | --- | --- | --- | --- | --- | --- | --- | --- | --- | --- | --- | --- | --- | --- | --- | --- | --- | --- | --- | --- | --- | --- | --- | --- | --- | --- | --- | --- | --- | --- | --- | --- | --- | --- | --- | --- | --- | --- | --- | --- | --- | --- | --- | --- | --- | --- | --- | --- | --- | --- | --- | --- | --- | --- | --- | --- | --- | --- | --- | --- | --- | --- | --- | --- | --- | --- | --- | --- | --- | --- | --- | --- | --- | --- | --- | --- | --- | --- | --- | --- | --- | --- | --- | --- | --- | --- | --- | --- | --- | --- | --- | --- | --- | --- | --- | --- | --- | --- | --- | --- | --- | --- | --- | --- | --- | --- | --- | --- | --- | --- | --- | --- | --- | --- | --- | --- | --- | --- | --- | --- | --- | --- | --- | --- | --- | --- |

| **Fisher's Exact Test** | |
| --- | --- |
| **Table Probability (P)** | <.0001 |
| **Pr <= P** | 0.5936 |

| **Sample Size = 73** |
| --- |

| \| \| **Frequency** \| \| --- \| \| **Percent** \| \| **Row Pct** \| \| **Col Pct** \| \| \| --- \| --- \| --- \| --- \| --- \| | \| **Table of Type by Final_group** \| \| \| \| \| \| \| --- \| --- \| --- \| --- \| --- \| --- \| \| **Type(Type)** \| **Final_group(Final_group)** \| \| \| \| \| \| **1** \| **2** \| **3** \| **4** \| **Total** \| \| **1** \| \| 2 \| \| --- \| \| 2.74 \| \| 20.00 \| \| 22.22 \| \| \| 7 \| \| --- \| \| 9.59 \| \| 70.00 \| \| 18.42 \| \| \| 0 \| \| --- \| \| 0.00 \| \| 0.00 \| \| 0.00 \| \| \| 1 \| \| --- \| \| 1.37 \| \| 10.00 \| \| 5.88 \| \| \| 10 \| \| --- \| \| 13.70 \| \|  \| \|  \| \| \| **2** \| \| 4 \| \| --- \| \| 5.48 \| \| 28.57 \| \| 44.44 \| \| \| 8 \| \| --- \| \| 10.96 \| \| 57.14 \| \| 21.05 \| \| \| 0 \| \| --- \| \| 0.00 \| \| 0.00 \| \| 0.00 \| \| \| 2 \| \| --- \| \| 2.74 \| \| 14.29 \| \| 11.76 \| \| \| 14 \| \| --- \| \| 19.18 \| \|  \| \|  \| \| \| **3** \| \| 3 \| \| --- \| \| 4.11 \| \| 6.12 \| \| 33.33 \| \| \| 23 \| \| --- \| \| 31.51 \| \| 46.94 \| \| 60.53 \| \| \| 9 \| \| --- \| \| 12.33 \| \| 18.37 \| \| 100.00 \| \| \| 14 \| \| --- \| \| 19.18 \| \| 28.57 \| \| 82.35 \| \| \| 49 \| \| --- \| \| 67.12 \| \|  \| \|  \| \| \| **Total** \| \| 9 \| \| --- \| \| 12.33 \| \| \| 38 \| \| --- \| \| 52.05 \| \| \| 9 \| \| --- \| \| 12.33 \| \| \| 17 \| \| --- \| \| 23.29 \| \| \| 73 \| \| --- \| \| 100.00 \| \| |
| --- | --- | --- | --- | --- | --- | --- | --- | --- | --- | --- | --- | --- | --- | --- | --- | --- | --- | --- | --- | --- | --- | --- | --- | --- | --- | --- | --- | --- | --- | --- | --- | --- | --- | --- | --- | --- | --- | --- | --- | --- | --- | --- | --- | --- | --- | --- | --- | --- | --- | --- | --- | --- | --- | --- | --- | --- | --- | --- | --- | --- | --- | --- | --- | --- | --- | --- | --- | --- | --- | --- | --- | --- | --- | --- | --- | --- | --- | --- | --- | --- | --- | --- | --- | --- | --- | --- | --- | --- | --- | --- | --- | --- | --- | --- | --- | --- | --- | --- | --- | --- | --- | --- | --- | --- | --- | --- | --- | --- | --- | --- | --- | --- | --- | --- | --- | --- | --- |

| **Fisher's Exact Test** | |
| --- | --- |
| **Table Probability (P)** | <.0001 |
| **Pr <= P** | 0.0580 |

| **Sample Size = 73** |
| --- |

| \| \| **Frequency** \| \| --- \| \| **Percent** \| \| **Row Pct** \| \| **Col Pct** \| \| \| --- \| --- \| --- \| --- \| --- \| | \| **Table of RA by Final_group** \| \| \| \| \| \| \| --- \| --- \| --- \| --- \| --- \| --- \| \| **RA(RA)** \| **Final_group(Final_group)** \| \| \| \| \| \| **1** \| **2** \| **3** \| **4** \| **Total** \| \| **0** \| \| 3 \| \| --- \| \| 4.11 \| \| 9.38 \| \| 33.33 \| \| \| 18 \| \| --- \| \| 24.66 \| \| 56.25 \| \| 47.37 \| \| \| 4 \| \| --- \| \| 5.48 \| \| 12.50 \| \| 44.44 \| \| \| 7 \| \| --- \| \| 9.59 \| \| 21.88 \| \| 41.18 \| \| \| 32 \| \| --- \| \| 43.84 \| \|  \| \|  \| \| \| **1** \| \| 6 \| \| --- \| \| 8.22 \| \| 15.00 \| \| 66.67 \| \| \| 20 \| \| --- \| \| 27.40 \| \| 50.00 \| \| 52.63 \| \| \| 5 \| \| --- \| \| 6.85 \| \| 12.50 \| \| 55.56 \| \| \| 9 \| \| --- \| \| 12.33 \| \| 22.50 \| \| 52.94 \| \| \| 40 \| \| --- \| \| 54.79 \| \|  \| \|  \| \| \| **2** \| \| 0 \| \| --- \| \| 0.00 \| \| 0.00 \| \| 0.00 \| \| \| 0 \| \| --- \| \| 0.00 \| \| 0.00 \| \| 0.00 \| \| \| 0 \| \| --- \| \| 0.00 \| \| 0.00 \| \| 0.00 \| \| \| 1 \| \| --- \| \| 1.37 \| \| 100.00 \| \| 5.88 \| \| \| 1 \| \| --- \| \| 1.37 \| \|  \| \|  \| \| \| **Total** \| \| 9 \| \| --- \| \| 12.33 \| \| \| 38 \| \| --- \| \| 52.05 \| \| \| 9 \| \| --- \| \| 12.33 \| \| \| 17 \| \| --- \| \| 23.29 \| \| \| 73 \| \| --- \| \| 100.00 \| \| |
| --- | --- | --- | --- | --- | --- | --- | --- | --- | --- | --- | --- | --- | --- | --- | --- | --- | --- | --- | --- | --- | --- | --- | --- | --- | --- | --- | --- | --- | --- | --- | --- | --- | --- | --- | --- | --- | --- | --- | --- | --- | --- | --- | --- | --- | --- | --- | --- | --- | --- | --- | --- | --- | --- | --- | --- | --- | --- | --- | --- | --- | --- | --- | --- | --- | --- | --- | --- | --- | --- | --- | --- | --- | --- | --- | --- | --- | --- | --- | --- | --- | --- | --- | --- | --- | --- | --- | --- | --- | --- | --- | --- | --- | --- | --- | --- | --- | --- | --- | --- | --- | --- | --- | --- | --- | --- | --- | --- | --- | --- | --- | --- | --- | --- | --- | --- | --- | --- |

| **Fisher's Exact Test** | |
| --- | --- |
| **Table Probability (P)** | 0.0033 |
| **Pr <= P** | 0.7661 |

|  | **Sample Size = 73** |
| --- | --- |

| The SAS System |
| --- |

The NPAR1WAY Procedure

| **Wilcoxon Scores (Rank Sums) for Variable MOI Classified by Variable Final_group** | | | | | |
| --- | --- | --- | --- | --- | --- |
| **Final_group** | **N** | **Sum of Scores** | **Expected Under H0** | **Std Dev Under H0** | **Mean Score** |
| **2** | 38 | 1379.0 | 1406.0 | 80.425045 | 36.289474 |
| **1** | 9 | 400.0 | 333.0 | 52.926933 | 44.444444 |
| **3** | 9 | 254.0 | 333.0 | 52.926933 | 28.222222 |
| **4** | 17 | 668.0 | 629.0 | 68.043080 | 39.294118 |
| **Average scores were used for ties.** | | | | | |

| **Kruskal-Wallis Test** | | |
| --- | --- | --- |
| **Chi-Square** | **DF** | **Pr > ChiSq** |
| 3.6642 | 3 | 0.3001 |


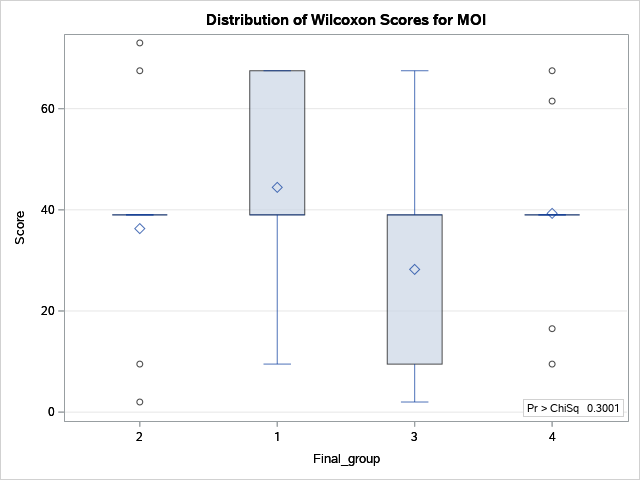


| The SAS System |
| --- |

The NPAR1WAY Procedure

| **Wilcoxon Scores (Rank Sums) for Variable ISS Classified by Variable Final_group** | | | | | |
| --- | --- | --- | --- | --- | --- |
| **Final_group** | **N** | **Sum of Scores** | **Expected Under H0** | **Std Dev Under H0** | **Mean Score** |
| **2** | 38 | 1260.00 | 1387.00 | 87.677963 | 33.157895 |
| **1** | 9 | 377.50 | 328.50 | 58.083226 | 41.944444 |
| **3** | 9 | 448.50 | 328.50 | 58.083226 | 49.833333 |
| **4** | 16 | 542.00 | 584.00 | 73.015187 | 33.875000 |
| **Average scores were used for ties.** | | | | | |

| **Kruskal-Wallis Test** | | |
| --- | --- | --- |
| **Chi-Square** | **DF** | **Pr > ChiSq** |
| 5.6057 | 3 | 0.1325 |


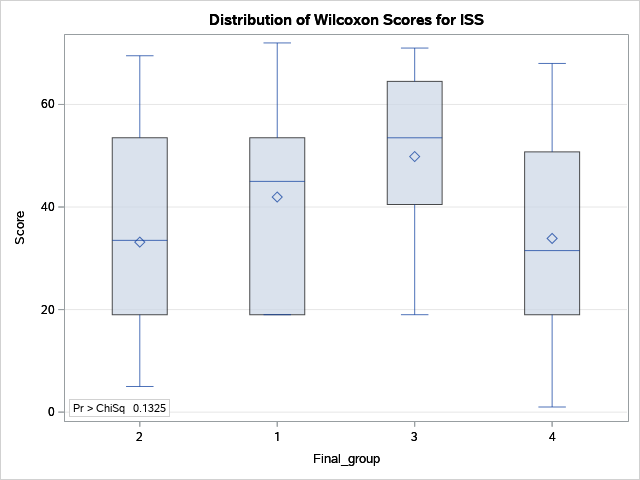


| The SAS System |
| --- |

The NPAR1WAY Procedure

| **Wilcoxon Scores (Rank Sums) for Variable LOS Classified by Variable Final_group** | | | | | |
| --- | --- | --- | --- | --- | --- |
| **Final_group** | **N** | **Sum of Scores** | **Expected Under H0** | **Std Dev Under H0** | **Mean Score** |
| **2** | 38 | 1296.00 | 1387.00 | 88.606997 | 34.105263 |
| **1** | 8 | 249.50 | 292.00 | 55.779131 | 31.187500 |
| **3** | 9 | 449.50 | 328.50 | 58.698674 | 49.944444 |
| **4** | 17 | 633.00 | 620.50 | 75.377645 | 37.235294 |
| **Average scores were used for ties.** | | | | | |

| **Kruskal-Wallis Test** | | |
| --- | --- | --- |
| **Chi-Square** | **DF** | **Pr > ChiSq** |
| 4.7532 | 3 | 0.1908 |


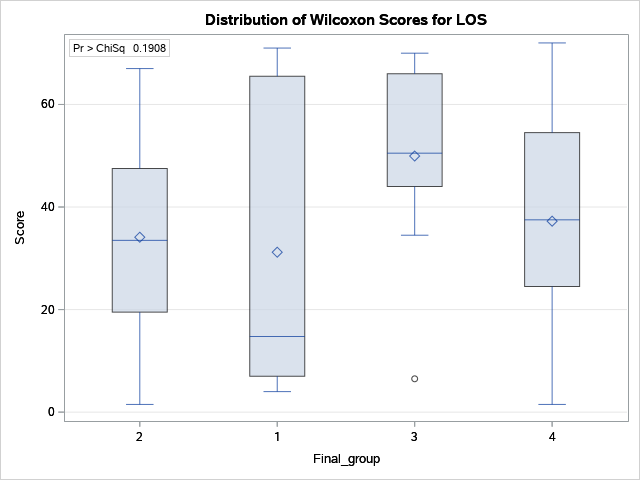


| The SAS System |
| --- |

The ANOVA Procedure

Dependent Variable: AGE AGE

| **Source** | **DF** | **Sum of Squares** | **Mean Square** | **F Value** | **Pr > F** |
| --- | --- | --- | --- | --- | --- |
| **Model** | 3 | 2027.88642 | 675.96214 | 2.09 | 0.1100 |
| **Error** | 69 | 22360.12728 | 324.05982 |  |  |
| **Corrected Total** | 72 | 24388.01370 |  |  |  |

| **R-Square** | **Coeff Var** | **Root MSE** | **AGE Mean** |
| --- | --- | --- | --- |
| 0.083151 | 28.51516 | 18.00166 | 63.13014 |

| **Source** | **DF** | **Anova SS** | **Mean Square** | **F Value** | **Pr > F** |
| --- | --- | --- | --- | --- | --- |
| **Final_group** | 3 | 2027.886420 | 675.962140 | 2.09 | 0.1100 |


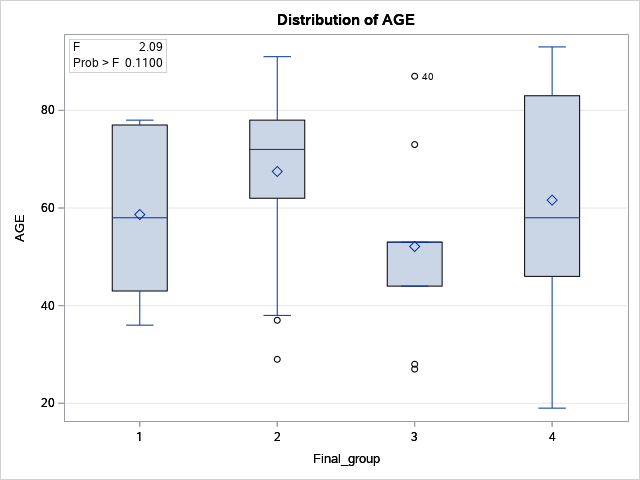

Supplement: Supplementary file 2 — Supplementary file2 (DOCX 97 KB) [file 11845_2024_3866_MOESM2_ESM.docx]
