# Supplementary material for: Insights into VTE risk in trauma patients: an observational study in an Irish trauma patient population
Source: Ir J Med Sci. 2025 Jan 17;194(1):195–204. doi: 10.1007/s11845-024-03866-4 (PMC11861230; doi:10.1007/s11845-024-03866-4)
Supplement: Supplementary file 3 — Supplementary file3 (DOCX 152 KB) [file 11845_2024_3866_MOESM3_ESM.docx]

| The SAS System |
| --- |

The TTEST Procedure

Variable: AGE (AGE)

| **VTEyesno** | **Method** | **N** | **Mean** | **Std Dev** | **Std Err** | **Minimum** | **Maximum** |
| --- | --- | --- | --- | --- | --- | --- | --- |
| **0** |  | 2807 | 60.0609 | 17.4526 | 0.3294 | 19.0000 | 93.0000 |
| **1** |  | 73 | 63.1301 | 18.4044 | 2.1541 | 19.0000 | 93.0000 |
| **Diff (1-2)** | **Pooled** |  | -3.0692 | 17.4770 | 2.0720 |  |  |
| **Diff (1-2)** | **Satterthwaite** |  | -3.0692 |  | 2.1791 |  |  |

| **VTEyesno** | **Method** | **Mean** | **95% CL Mean** | | **Std Dev** | **95% CL Std Dev** | |
| --- | --- | --- | --- | --- | --- | --- | --- |
| **0** |  | 60.0609 | 59.4150 | 60.7068 | 17.4526 | 17.0077 | 17.9215 |
| **1** |  | 63.1301 | 58.8361 | 67.4242 | 18.4044 | 15.8275 | 21.9914 |
| **Diff (1-2)** | **Pooled** | -3.0692 | -7.1319 | 0.9935 | 17.4770 | 17.0370 | 17.9405 |
| **Diff (1-2)** | **Satterthwaite** | -3.0692 | -7.4099 | 1.2714 |  |  |  |

| **Method** | **Variances** | **DF** | **t Value** | **Pr > \|t\|** |
| --- | --- | --- | --- | --- |
| **Pooled** | Equal | 2878 | -1.48 | 0.1386 |
| **Satterthwaite** | Unequal | 75.406 | -1.41 | 0.1631 |

| **Equality of Variances** | | | | |
| --- | --- | --- | --- | --- |
| **Method** | **Num DF** | **Den DF** | **F Value** | **Pr > F** |
| **Folded F** | 72 | 2806 | 1.11 | 0.4884 |


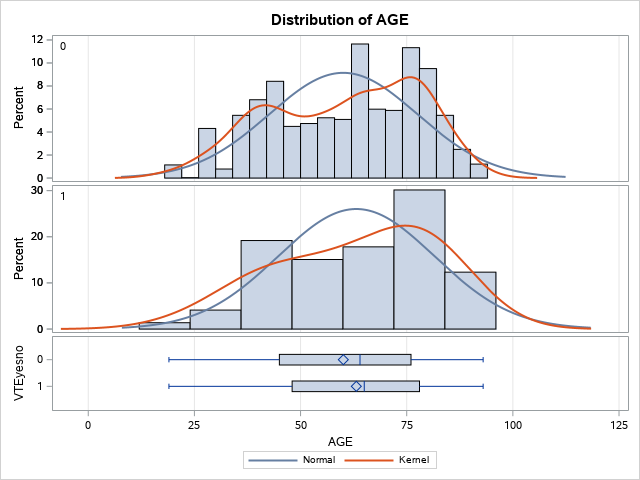


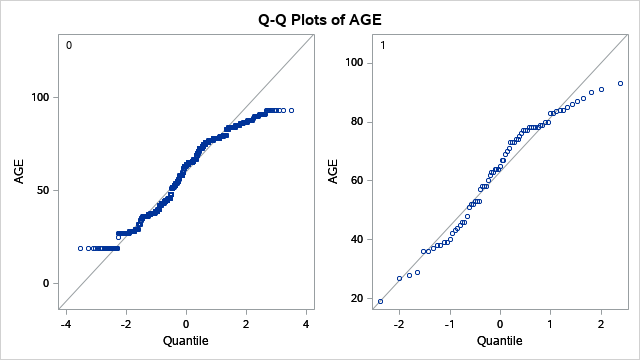


| The SAS System |
| --- |

The NPAR1WAY Procedure

| **Wilcoxon Scores (Rank Sums) for Variable MOI Classified by Variable VTEyesno** | | | | | |
| --- | --- | --- | --- | --- | --- |
| **VTEyesno** | **N** | **Sum of Scores** | **Expected Under H0** | **Std Dev Under H0** | **Mean Score** |
| **1** | 73 | 101664.50 | 104974.0 | 6495.16856 | 1392.66438 |
| **0** | 2802 | 4032585.50 | 4029276.0 | 6495.16856 | 1439.18112 |
| **Average scores were used for ties.** | | | | | |

| **Wilcoxon Two-Sample Test** | | | | | |
| --- | --- | --- | --- | --- | --- |
| **Statistic** | **Z** | **Pr < Z** | **Pr > \|Z\|** | **t Approximation** | |
|  |  |  |  | **Pr < Z** | **Pr > \|Z\|** |
| 101664.5 | -0.5095 | 0.3052 | 0.6104 | 0.3052 | 0.6105 |
| **Z includes a continuity correction of 0.5.** | | | | | |

| **Kruskal-Wallis Test** | | |
| --- | --- | --- |
| **Chi-Square** | **DF** | **Pr > ChiSq** |
| 0.2596 | 1 | 0.6104 |


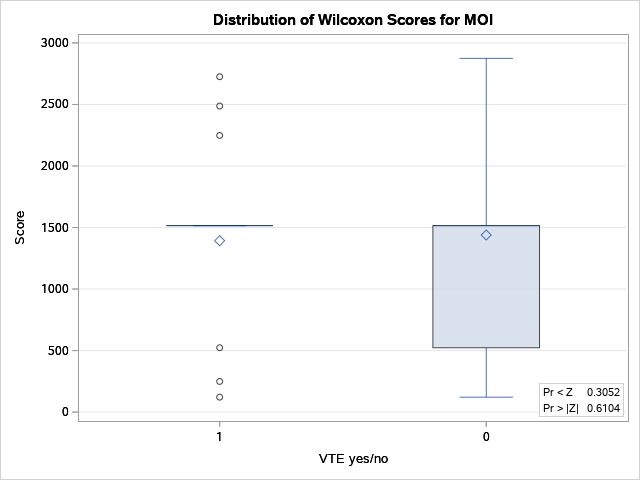


| The SAS System |
| --- |

The NPAR1WAY Procedure

| **Wilcoxon Scores (Rank Sums) for Variable ISS Classified by Variable VTEyesno** | | | | | |
| --- | --- | --- | --- | --- | --- |
| **VTEyesno** | **N** | **Sum of Scores** | **Expected Under H0** | **Std Dev Under H0** | **Mean Score** |
| **1** | 72 | 119804.50 | 103500.0 | 6867.29125 | 1663.95139 |
| **0** | 2802 | 4011570.50 | 4027875.0 | 6867.29125 | 1431.68112 |
| **Average scores were used for ties.** | | | | | |

| **Wilcoxon Two-Sample Test** | | | | | |
| --- | --- | --- | --- | --- | --- |
| **Statistic** | **Z** | **Pr > Z** | **Pr > \|Z\|** | **t Approximation** | |
|  |  |  |  | **Pr > Z** | **Pr > \|Z\|** |
| 119804.5 | 2.3742 | 0.0088 | 0.0176 | 0.0088 | 0.0177 |
| **Z includes a continuity correction of 0.5.** | | | | | |

| **Kruskal-Wallis Test** | | |
| --- | --- | --- |
| **Chi-Square** | **DF** | **Pr > ChiSq** |
| 5.6369 | 1 | 0.0176 |


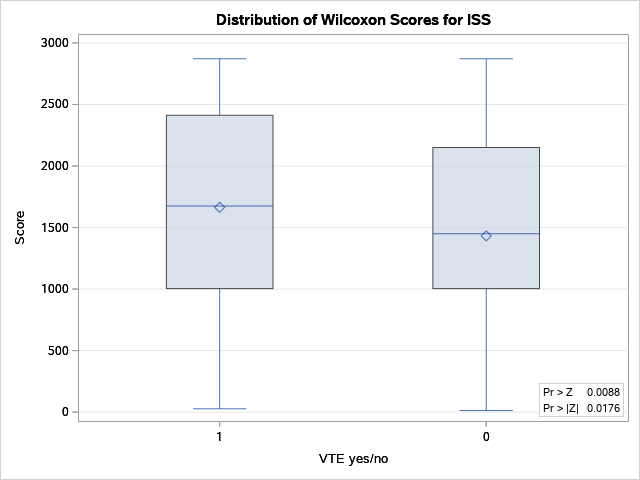


| The SAS System |
| --- |

The NPAR1WAY Procedure

| **Wilcoxon Scores (Rank Sums) for Variable LOS Classified by Variable VTEyesno** | | | | | |
| --- | --- | --- | --- | --- | --- |
| **VTEyesno** | **N** | **Sum of Scores** | **Expected Under H0** | **Std Dev Under H0** | **Mean Score** |
| **1** | 72 | 133617.50 | 103500.0 | 6942.53319 | 1855.79861 |
| **0** | 2802 | 3997757.50 | 4027875.0 | 6942.53319 | 1426.75143 |
| **Average scores were used for ties.** | | | | | |

| **Wilcoxon Two-Sample Test** | | | | | |
| --- | --- | --- | --- | --- | --- |
| **Statistic** | **Z** | **Pr > Z** | **Pr > \|Z\|** | **t Approximation** | |
|  |  |  |  | **Pr > Z** | **Pr > \|Z\|** |
| 133617.5 | 4.3380 | <.0001 | <.0001 | <.0001 | <.0001 |
| **Z includes a continuity correction of 0.5.** | | | | | |

| **Kruskal-Wallis Test** | | |
| --- | --- | --- |
| **Chi-Square** | **DF** | **Pr > ChiSq** |
| 18.8192 | 1 | <.0001 |


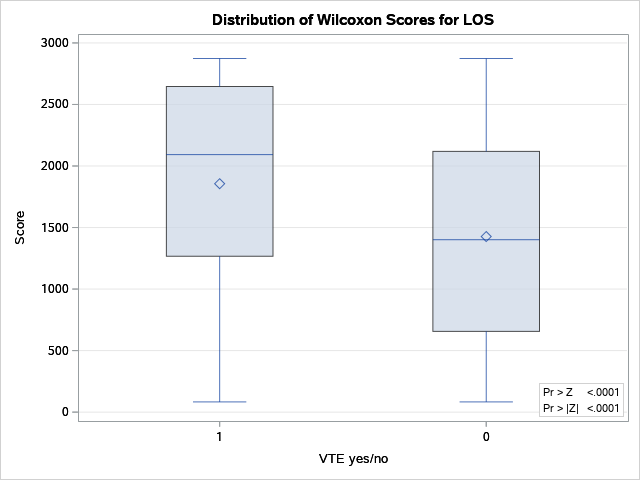


| The SAS System |
| --- |

The FREQ Procedure

| \| \| **Frequency** \| \| --- \| \| **Percent** \| \| **Row Pct** \| \| **Col Pct** \| \| \| --- \| --- \| --- \| --- \| --- \| | \| **Table of gender by VTEyesno** \| \| \| \| \| --- \| --- \| --- \| --- \| \| **gender(gender)** \| **VTEyesno(VTE yes/no)** \| \| \| \| **0** \| **1** \| **Total** \| \| **0** \| \| 1812 \| \| --- \| \| 62.94 \| \| 97.05 \| \| 64.58 \| \| \| 55 \| \| --- \| \| 1.91 \| \| 2.95 \| \| 75.34 \| \| \| 1867 \| \| --- \| \| 64.85 \| \|  \| \|  \| \| \| **1** \| \| 994 \| \| --- \| \| 34.53 \| \| 98.22 \| \| 35.42 \| \| \| 18 \| \| --- \| \| 0.63 \| \| 1.78 \| \| 24.66 \| \| \| 1012 \| \| --- \| \| 35.15 \| \|  \| \|  \| \| \| **Total** \| \| 2806 \| \| --- \| \| 97.46 \| \| \| 73 \| \| --- \| \| 2.54 \| \| \| 2879 \| \| --- \| \| 100.00 \| \| \| **Frequency Missing = 1** \| \| \| \| |
| --- | --- | --- | --- | --- | --- | --- | --- | --- | --- | --- | --- | --- | --- | --- | --- | --- | --- | --- | --- | --- | --- | --- | --- | --- | --- | --- | --- | --- | --- | --- | --- | --- | --- | --- | --- | --- | --- | --- | --- | --- | --- | --- | --- | --- | --- | --- | --- | --- | --- | --- | --- | --- | --- | --- | --- | --- | --- | --- | --- | --- | --- | --- | --- |

| **Statistics for Table of gender by VTEyesno** |
| --- |

| **Statistic** | **DF** | **Value** | **Prob** |
| --- | --- | --- | --- |
| **Chi-Square** | 1 | 3.6181 | 0.0572 |
| **Likelihood Ratio Chi-Square** | 1 | 3.8252 | 0.0505 |
| **Continuity Adj. Chi-Square** | 1 | 3.1612 | 0.0754 |
| **Mantel-Haenszel Chi-Square** | 1 | 3.6168 | 0.0572 |
| **Phi Coefficient** |  | -0.0355 |  |
| **Contingency Coefficient** |  | 0.0354 |  |
| **Cramer's V** |  | -0.0355 |  |

| **Fisher's Exact Test** | |
| --- | --- |
| **Cell (1,1) Frequency (F)** | 1812 |
| **Left-sided Pr <= F** | 0.0352 |
| **Right-sided Pr >= F** | 0.9809 |
|  |  |
| **Table Probability (P)** | 0.0161 |
| **Two-sided Pr <= P** | 0.0625 |

| **Sample Size = 2879 Frequency Missing = 1** |
| --- |

| \| \| **Frequency** \| \| --- \| \| **Percent** \| \| **Row Pct** \| \| **Col Pct** \| \| \| --- \| --- \| --- \| --- \| --- \| | \| **Table of dead by VTEyesno** \| \| \| \| \| --- \| --- \| --- \| --- \| \| **dead(dead)** \| **VTEyesno(VTE yes/no)** \| \| \| \| **0** \| **1** \| **Total** \| \| **0** \| \| 2531 \| \| --- \| \| 87.94 \| \| 97.68 \| \| 90.20 \| \| \| 60 \| \| --- \| \| 2.08 \| \| 2.32 \| \| 83.33 \| \| \| 2591 \| \| --- \| \| 90.03 \| \|  \| \|  \| \| \| **1** \| \| 275 \| \| --- \| \| 9.56 \| \| 95.82 \| \| 9.80 \| \| \| 12 \| \| --- \| \| 0.42 \| \| 4.18 \| \| 16.67 \| \| \| 287 \| \| --- \| \| 9.97 \| \|  \| \|  \| \| \| **Total** \| \| 2806 \| \| --- \| \| 97.50 \| \| \| 72 \| \| --- \| \| 2.50 \| \| \| 2878 \| \| --- \| \| 100.00 \| \| \| **Frequency Missing = 2** \| \| \| \| |
| --- | --- | --- | --- | --- | --- | --- | --- | --- | --- | --- | --- | --- | --- | --- | --- | --- | --- | --- | --- | --- | --- | --- | --- | --- | --- | --- | --- | --- | --- | --- | --- | --- | --- | --- | --- | --- | --- | --- | --- | --- | --- | --- | --- | --- | --- | --- | --- | --- | --- | --- | --- | --- | --- | --- | --- | --- | --- | --- | --- | --- | --- | --- | --- |

| **Statistics for Table of dead by VTEyesno** |
| --- |

| **Statistic** | **DF** | **Value** | **Prob** |
| --- | --- | --- | --- |
| **Chi-Square** | 1 | 3.6864 | 0.0549 |
| **Likelihood Ratio Chi-Square** | 1 | 3.1469 | 0.0761 |
| **Continuity Adj. Chi-Square** | 1 | 2.9612 | 0.0853 |
| **Mantel-Haenszel Chi-Square** | 1 | 3.6851 | 0.0549 |
| **Phi Coefficient** |  | 0.0358 |  |
| **Contingency Coefficient** |  | 0.0358 |  |
| **Cramer's V** |  | 0.0358 |  |

| **Fisher's Exact Test** | |
| --- | --- |
| **Cell (1,1) Frequency (F)** | 2531 |
| **Left-sided Pr <= F** | 0.9767 |
| **Right-sided Pr >= F** | 0.0498 |
|  |  |
| **Table Probability (P)** | 0.0265 |
| **Two-sided Pr <= P** | 0.0697 |

| **Sample Size = 2878 Frequency Missing = 2** |
| --- |

**VTE groups only (the missing comparisons!)**

| The SAS System |
| --- |

The NPAR1WAY Procedure

| **Wilcoxon Scores (Rank Sums) for Variable Daystoprophy Classified by Variable Final_group** | | | | | |
| --- | --- | --- | --- | --- | --- |
| **Final_group** | **N** | **Sum of Scores** | **Expected Under H0** | **Std Dev Under H0** | **Mean Score** |
| **2** | 25 | 673.00 | 637.50 | 45.574563 | 26.920000 |
| **1** | 4 | 118.00 | 102.00 | 24.728138 | 29.500000 |
| **3** | 7 | 209.50 | 178.50 | 31.627565 | 29.928571 |
| **4** | 14 | 274.50 | 357.00 | 40.925856 | 19.607143 |
| **Average scores were used for ties.** | | | | | |

| **Kruskal-Wallis Test** | | |
| --- | --- | --- |
| **Chi-Square** | **DF** | **Pr > ChiSq** |
| 4.4406 | 3 | 0.2177 |


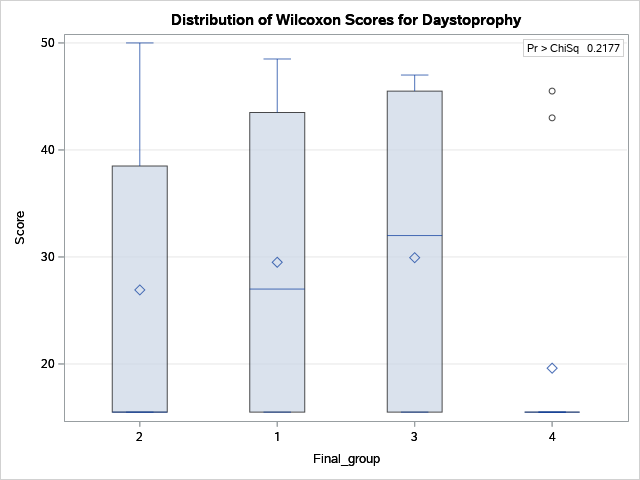


| The SAS System |
| --- |

The NPAR1WAY Procedure

| **Pairwise Two-Sided Multiple Comparison Analysis** | | | |
| --- | --- | --- | --- |
| **Dwass, Steel, Critchlow-Fligner Method** | | | |
| **Variable: Daystoprophy** | | | |
| **Final_group** | **Wilcoxon Z** | **DSCF Value** | **Pr > DSCF** |
| **2 vs. 1** | -0.3759 | 0.5317 | 0.9819 |
| **2 vs. 3** | -0.5374 | 0.7600 | 0.9499 |
| **2 vs. 4** | 1.7755 | 2.5109 | 0.2852 |
| **1 vs. 3** | 0.0994 | 0.1405 | 0.9996 |
| **1 vs. 4** | 1.4581 | 2.0621 | 0.4630 |
| **3 vs. 4** | 1.9182 | 2.7127 | 0.2203 |

| The SAS System |
| --- |

The FREQ Procedure

| \| \| **Frequency** \| \| --- \| \| **Percent** \| \| **Row Pct** \| \| **Col Pct** \| \| \| --- \| --- \| --- \| --- \| --- \| | \| **Table of Receivedprophylaxis by Final_group** \| \| \| \| \| \| \| --- \| --- \| --- \| --- \| --- \| --- \| \| **Receivedprophylaxis(Received prophylaxis)** \| **Final_group(Final_group)** \| \| \| \| \| \| **1** \| **2** \| **3** \| **4** \| **Total** \| \| **0** \| \| 5 \| \| --- \| \| 6.85 \| \| 21.74 \| \| 55.56 \| \| \| 13 \| \| --- \| \| 17.81 \| \| 56.52 \| \| 34.21 \| \| \| 2 \| \| --- \| \| 2.74 \| \| 8.70 \| \| 22.22 \| \| \| 3 \| \| --- \| \| 4.11 \| \| 13.04 \| \| 17.65 \| \| \| 23 \| \| --- \| \| 31.51 \| \|  \| \|  \| \| \| **1** \| \| 4 \| \| --- \| \| 5.48 \| \| 8.00 \| \| 44.44 \| \| \| 25 \| \| --- \| \| 34.25 \| \| 50.00 \| \| 65.79 \| \| \| 7 \| \| --- \| \| 9.59 \| \| 14.00 \| \| 77.78 \| \| \| 14 \| \| --- \| \| 19.18 \| \| 28.00 \| \| 82.35 \| \| \| 50 \| \| --- \| \| 68.49 \| \|  \| \|  \| \| \| **Total** \| \| 9 \| \| --- \| \| 12.33 \| \| \| 38 \| \| --- \| \| 52.05 \| \| \| 9 \| \| --- \| \| 12.33 \| \| \| 17 \| \| --- \| \| 23.29 \| \| \| 73 \| \| --- \| \| 100.00 \| \| |
| --- | --- | --- | --- | --- | --- | --- | --- | --- | --- | --- | --- | --- | --- | --- | --- | --- | --- | --- | --- | --- | --- | --- | --- | --- | --- | --- | --- | --- | --- | --- | --- | --- | --- | --- | --- | --- | --- | --- | --- | --- | --- | --- | --- | --- | --- | --- | --- | --- | --- | --- | --- | --- | --- | --- | --- | --- | --- | --- | --- | --- | --- | --- | --- | --- | --- | --- | --- | --- | --- | --- | --- | --- | --- | --- | --- | --- | --- | --- | --- | --- | --- | --- | --- | --- | --- | --- | --- | --- | --- | --- | --- |

| **Statistics for Table of Receivedprophylaxis by Final_group** |
| --- |

| **Statistic** | **DF** | **Value** | **Prob** |
| --- | --- | --- | --- |
| **Chi-Square** | 3 | 4.4135 | 0.2201 |
| **Likelihood Ratio Chi-Square** | 3 | 4.4040 | 0.2210 |
| **Mantel-Haenszel Chi-Square** | 1 | 3.8745 | 0.0490 |
| **Phi Coefficient** |  | 0.2459 |  |
| **Contingency Coefficient** |  | 0.2388 |  |
| **Cramer's V** |  | 0.2459 |  |
| **WARNING: 25% of the cells have expected counts less  than 5. Chi-Square may not be a valid test.** | | | |

| **Fisher's Exact Test** | |
| --- | --- |
| **Table Probability (P)** | 0.0029 |
| **Pr <= P** | 0.2412 |

| **Sample Size = 73** |
| --- |
